# Cannonball Metastatic Lesions in a Young Male: A Case Report

**DOI:** 10.14740/wjon786w

**Published:** 2014-05-06

**Authors:** Zubin Arora, Abhijit Duggal

**Affiliations:** a Department of Internal Medicine, Cleveland Clinic, Cleveland, OH 44195, USA; b Medical Intensive Care Unit, Respiratory Institute, Cleveland Clinic, Cleveland, OH 44195, USA

**Keywords:** Cannonball lesions, Drug abuse, Germ cell tumor, Metastases, Metastatic testicular cancer

## Abstract

Testicular germ cell tumors are the most common malignancy among young men. These are highly chemo-sensitive tumors with high cure rates. More than 95% of patients with testicular cancer present with a painless testicular mass. Here we describe a rare initial presentation of testicular cancer in a previously asymptomatic 22-year-old male who presented with widespread metastatic cannonball lesions in his lungs.

## Introduction

Testicular cancer is one of the most common malignancies which affect young men and its incidence appears to be increasing with time [1]. It presents as a painless testicular mass in more than 95% of the cases [2]. We describe an unusual initial presentation of testicular germ cell tumor in a previously asymptomatic young male who presented with metastatic disease to the lungs.

## Case Report

A 22-year-old male with a history of ongoing IV heroin abuse presented to the hospital with a cough productive of reddish sputum and shortness of breath of 4 days duration. He denied fever, chills or other systemic symptoms. Past medical history was significant for a recent episode of left arm cellulitis around 2 months earlier which had resolved after treatment with amoxicillin-clavulanate. He had been seen several times in the outpatient and inpatient setting in the past for substance abuse where attempts at detoxification and rehabilitation had been unsuccessful.

The patient was in respiratory distress on presentation, and his vital signs revealed oxygen saturation of 85%, blood pressure of 170/95 mmHg, pulse rate of 99/min and respiratory rate of 25/min. Physical exam showed track marks in the left antecubital fossa and bilateral coarse rhonchi in all lung fields. The patient was emergently intubated and admitted to the intensive care unit for management of respiratory failure.

**Figure 1:**
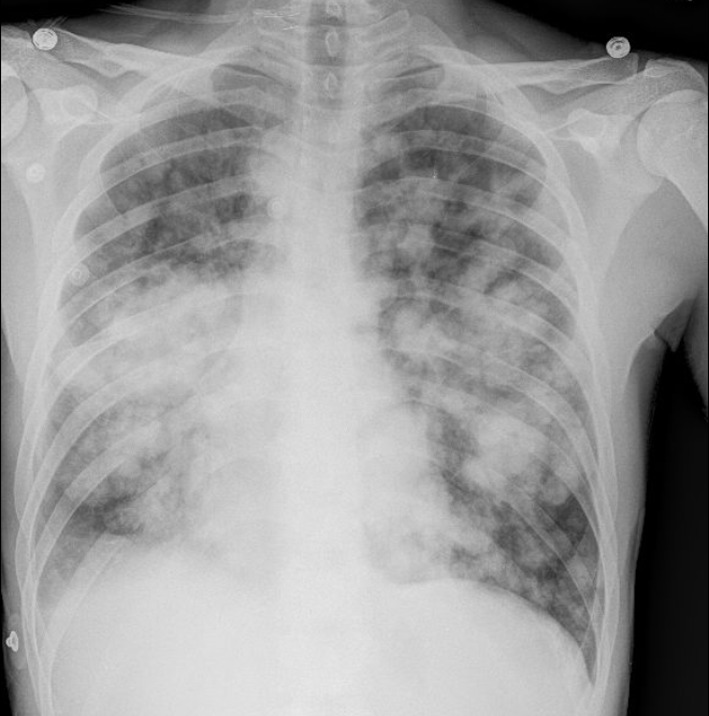
Chest X-ray showing bilateral rounded airspace opacities.

**Figure 2:**
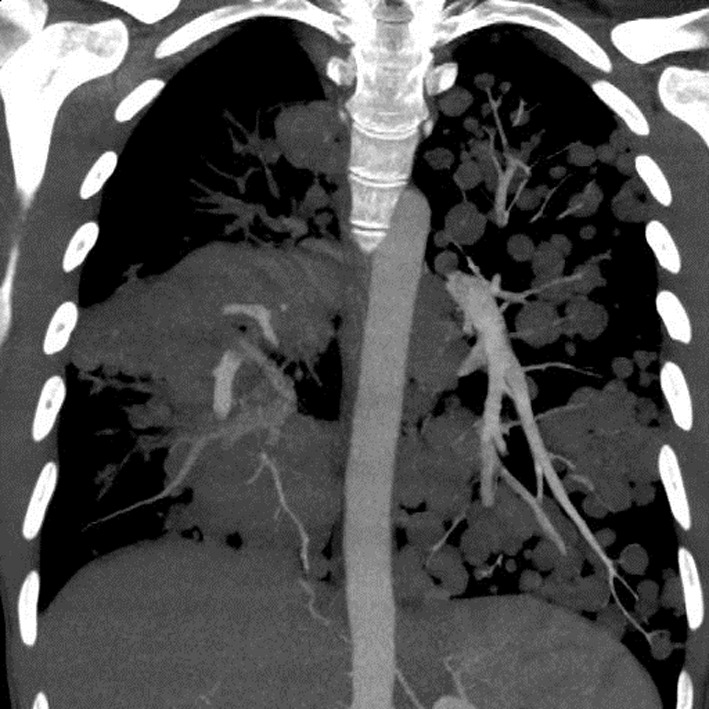
Coronal section of CT chest showing bilateral cannonball pulmonary nodules.

**Figure 3:**
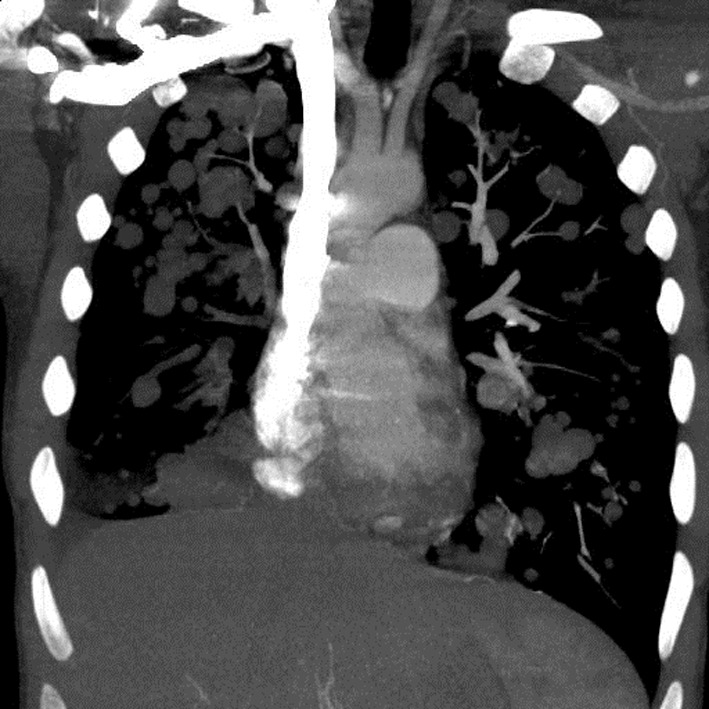
Coronal section of CT chest showing bilateral cannonball pulmonary nodules.

A chest X-ray (Fig. 1) was obtained and showed the presence of multifocal rounded airspace opacities within both lungs. Subsequently, a CT scan of the chest (Fig. 2, 3) was obtained which demonstrated bilateral pulmonary emboli along with bilateral conglomerate pulmonary nodules in both lower lungs.

Based on patient’s history of IV drug abuse, recent episode of cellulitis, concomitant lower back pain and consistent imaging findings, the diagnosis of right-sided infective endocarditis with septic pulmonary embolism was initially considered and patient was empirically started on IV antibiotics after drawing blood cultures. However, a transthoracic echocardiogram obtained the next day did not reveal any vegetations and the patient continued to remain afebrile with all blood cultures coming back negative. This prompted a reconsideration of the diagnosis. All radiographic images were reviewed again with radiology and were notable for the lack of necrosis in the pulmonary nodules. A follow-up abdominal CT also revealed the presence of retroperitoneal lymphadenopathy indicating a possible neoplastic process.

A thorough physical exam was performed again which revealed the presence of a small left testicular mass. Ultrasound of the testes was obtained which showed two complex left testicular masses containing cystic and solid components as well as microcalcifications. His serum alpha fetoprotein, lactate dehydrogenase and beta-human chorionic gonadotropin were all found to be elevated.

The patient was diagnosed with metastatic non-seminomatous germ cell tumor of the testis. Because of the large tumor burden, he was started on chemotherapy with cisplatin, etoposide and iphosphamide, and orchiectomy was deferred for a later stage when he was more stable. He was extubated after receiving his first cycle of chemotherapy, and underwent left orchiectomy and retroperitoneal lymph node dissection soon after, with biopsy confirming the diagnosis of germ cell tumor with residual viable teratoma. He continues to receive medical care for malignancy as well as for substance use disorder as an outpatient.

## Discussion

Testicular germ cell tumors are the most frequent cause of malignancy in young males [1]. Despite its increasing incidence, mortality rate from testicular cancer continues to remain low [1, 2]. This is due to introduction of highly effective platinum-based chemotherapy regimens and the chemo-sensitive nature of the tumor. Even with metastatic disease, cure rates as high as 80% have been reported with the use of modern chemotherapy regimens [3]. In most patients, testicular cancer presents as a painless lump in the testis noticed incidentally by the patient. Due to the absence of pain, there can often be a delay in seeking medical care which may lead to metastatic spread of the disease by the time of presentation, thus affecting prognosis [4].

In rare cases, patients may present with symptoms from the metastasis. In these patients, a careful physical examination is of prime importance in pointing towards the correct diagnosis. Radiological features such as multiple lung nodules greater than 1 cm in size seen on a plain chest radiograph are also usually indicative of disseminated malignancy to the lungs [5, 6]. Such large, rounded “cannonball” nodules are associated with poor prognosis [7, 8].

Scrotal ultrasound is highly sensitive for detecting testicular masses and is the imaging of choice for confirming the presence of testicular tumors [9]. Blood concentrations of tumor markers including alpha fetoprotein and beta-human chorionic gonadotropin are often elevated in patients with testicular germ cell tumors. The levels of these markers should fall after treatment with orchiectomy and/or chemotherapy. Persisting or rising levels after therapy are indicative of residual tumor or recurrence.

This case highlights several important clinical points. First, it illustrates a rare initial presentation of testicular cancer with impressive radiological findings. Our patient was unaware of the presence of a tumor and his presenting symptoms were all related to the metastases. Second, it emphasizes the importance of thorough history taking, meticulous physical examination and broad differential diagnosis, the three pillars of practice of medicine. A careful urogenital examination during previous visits could have revealed the presence of the testicular mass, thus avoiding delay in diagnosis and initiation of treatment. Finally, it underscores the importance of a multidisciplinary team approach in the treatment of cancer patients. In the present case, several teams including pulmonology, oncology, urology, radiology, pathology and psychiatry, each contributed to the medical care of our patient.
